# Targeting hepatocyte-specific *SLC2A8* blocks hepatic steatosis and dissociates TCA cycle flux inhibition from glutamine anaplerosis

**DOI:** 10.1097/HC9.0000000000000810

**Published:** 2025-09-22

**Authors:** Joshua A. Adams, Yiming Zhang, Jiameng Sun, Andrew Tilston-Lunel, Cassandra B. Higgins, Monique Heitmeier, Sam Ballentine, Eric Tycksen, Roland E. Dolle, Paul W. Hruz, Brian J. DeBosch

**Affiliations:** 1 Department of Pediatrics, Indiana University School of Medicine, Indianapolis, Indiana, USA; 2 Department of Pediatrics, Washington University School of Medicine, St. Louis, Missouri, USA; 3 Department of Pathology & Immunology, Washington University School of Medicine, St. Louis, Missouri, USA; 4 McDonnell Genome Institute, Department of Genetics, Washington University School of Medicine, St. Louis, MO, USA; 5 Department of Biochemistry and Molecular Biophysics, Washington University School of Medicine, St. Louis, Missouri, USA; 6 Department of Pediatrics, Center for Nutrition and Molecular Metabolism, Herman B Wells Center for Pediatric Research, Indiana University School of Medicine, Indianapolis, Indiana, USA

**Keywords:** caloric restriction, energy metabolism, fasting, fructose, glucose transporter, GLUT8, glutamine anaplerosis, metabolic dysfunction–associated steatohepatitis, metabolic dysfunction–associated steatotic liver disease, TCA cycle

## Abstract

**Background:**

Excess TCA cycle and glutamine anaplerosis are hallmarks of metabolic dysfunction–associated steatotic liver disease and steatohepatitis. Blocking glutamine metabolism attenuates metabolic dysfunction–associated steatohepatitis. However, inhibiting TCA cycle flux by blocking plasma membrane carbohydrate transport is limited by the ubiquitous tissue distribution, function, and homology among the SLC2A family of facilitative carbohydrate transporters, and the potential for carbohydrate blockade to invoke or exacerbate glutamine anaplerosis. Here, we quantify alterations in hepatocyte carbon flux, define the broader metabolic consequences of hepatocyte-specific GLUT8/*SLC2A8* inhibition, and delineate the antisteatotic efficacy of a novel small-molecule GLUT8-selective inhibitor.

**Methods:**

We generated mice with floxed *SLC2A8* alleles and expressed hepatocyte-specific Cre by breeding these mice with albumin-Cre transgenic mice, or by administering AAV8 encoding hepatocyte-specific iCre. We performed stable-isotope glucose, fructose, and glutamine metabolic labeling in isolated GLUT8^WT^ and GLUT8^LKO^ hepatocytes and performed metabolic phenotyping in lean and diet-induced obese GLUT8^WT^ and GLUT8^LKO^ mice. Finally, we performed high-throughput screening to identify a GLUT8-selective inhibitor, which we characterized using in vitro models of triglyceride accumulation.

**Results:**

Hepatocyte-specific *SLC2A8* deletion reduced diet-induced hepatic and peripheral fat accumulation and increased thermogenesis during ZT12-24 (eg, the dark phase). It also disrupted TCA cycle flux without inducing compensatory glutamine utilization. High-throughput screening identified a small-molecule, GLUT8-selective inhibitor, P20, which blocked hepatocyte TG accumulation and inflammation in in vitro steatotic and inflammatory models.

**Conclusions:**

Deleting the hepatocyte carbohydrate transporter GLUT8 suppresses TCA cycle flux without inducing compensatory glutamine anaplerosis. The net effect of this is liver protection against multiple forms of dietary insult. Given that selective pharmacological GLUT8 inhibition is feasible, GLUT8 may be a viable target to abate metabolic dysfunction–associated steatohepatitis and other complications of obesity.

## Graphical abstract

**Figure:**
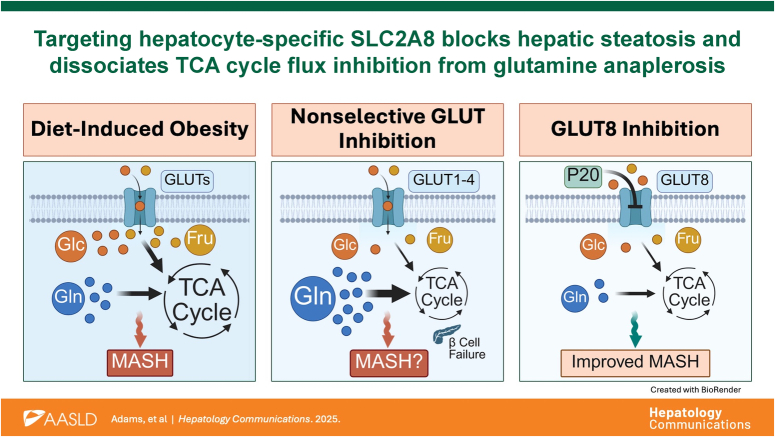

## INTRODUCTION

Carbohydrates carry high-energy carbon bonds to provide substrate for growth, proliferation, or storage as lipid or glycogen. The first and limiting step in carbohydrate catabolism is facilitative transport into the cell through the glucose transporter (GLUT) family of carbohydrate and polyol transporters.^1^ Recent data indicate that restricting carbohydrate entry through the GLUTs can abate metabolic disease.^1^^–^^7^ However, nonselective GLUT targeting can be detrimental to the organism.^8^^–^^10^ For example, blocking GLUT1 causes GLUT1 deficiency syndrome, which presents with seizures and neurologic deficits. Hepatocyte-specific GLUT2 deletion induces beta cell dysfunction and disrupts hepatic bile acid signaling.^8^ GLUT4 inhibition results in peripheral glucose intolerance. Therefore, isoform- and compartment-selective GLUT targeting is critical to optimally leverage this biology to abate overnutrition and tumor biology.

Prior work identified the facilitative glucose, fructose, trehalose, and galactose transporter, GLUT8 (encoded by the *Slc2a8* gene), as a target transporter that mediates growth and survival in solid and hematogenous tumors.^3^^,^^11^ Consistent with this, GLUT8 expression, localization, and function are deranged in diabetic models^12^^–^^14^ and in human malignancies.^3^^,^^11^^,^^15^ Moreover, germline GLUT8 deletion and antisense-mediated GLUT8 knockdown enhance thermogenesis and prevent diet-induced hepatic steatosis.^7^^,^^16^^–^^18^ Part of the mechanistic action of GLUT8 blockade is consistent with hepatocyte fasting-like signaling.^7^ For example, GLUT8 inhibition activates hepatocyte autophagic flux,^2^^,^^19^^–^^21^ and PPARα-FGF21 signaling.^5^ Inhibiting carbohydrate entry in a compartment- and isoform-specific manner to abate disease is thus an emerging concept toward precision therapy against cancer, metabolic, and inflammatory diseases.^15^^,^^22^ The liver is particularly amenable to GLUT targeting, in light of its positioning in first-pass metabolism of portal macronutrients, and its dynamic ability to coordinate the moment-to-moment fed and fasting state of the organism.

GLUT2 is the most abundantly expressed hepatic GLUT, and it mediates bile acid signaling and pancreatic islet beta cell competence.^8^ Indeed, hepatocyte-specific GLUT2 deletion in mice resulted in early-onset pancreatic beta cell failure.^8^ This indicated that GLUT2 inhibition in hepatocytes is not an optimal therapeutic strategy. In contrast, GLUT8 is the second most abundantly expressed hepatocyte GLUT in humans and mice.^17^ GLUT8 is expressed in several tissues, but is predominantly expressed in the testis, brain, and liver.^23^ Nevertheless, whole-body germline GLUT8 deletion and acute knockdown models indicate that whole-body GLUT8 blockade does not yield basal metabolic abnormalities, and GLUT8 inhibition attenuates diet-induced insulin resistance and hepatic steatosis.^7^^,^^16^^–^^18^ Furthermore, increased hepatic GLUT8 expression during high nutrient demand, such as during development, the fasted state,^7^ or nutrient overload,^12^ suggests that hepatic GLUT8 is an important metabolic regulator, a carbohydrate sensor, or both. Together, these findings prompted the overarching hypothesis that hepatocyte-specific GLUT8 is a tractable target to prevent and reverse metabolic liver disease.

Here, we established the metabolic consequences of hepatocyte GLUT8 blockade in vitro and in vivo. We first focused on the physiology of energy expenditure and glucose homeostasis in hepatocyte-specific GLUT8-deficient (GLUT8^LKO^) mice. We then extended these analyses toward a detailed view of carbon flux in GLUT8^LKO^ hepatocytes. This was an important set of analyses because (a) increased TCA cycle flux and glutamine anaplerosis are hallmarks of steatotic liver disease,^24^ and (b) nonselectively inhibiting carbohydrate metabolism through the class I GLUTs (GLUT1, 2, 3, and 4) provokes glutamine anaplerosis.^25^ Therefore, selectively inhibiting TCA cycle without provoking glutamine anaplerosis in treating metabolic dysfunction–associated steatohepatitis (MASH) may critically advance the therapeutic potential for GLUT inhibitors.

We provide evidence that hepatocyte-specific GLUT8 deletion impairs glucose and fructose-mediated hepatocyte TCA cycle flux. Importantly, TCA cycle inhibition was independent of glutamine anaplerosis. At the level of systems physiology, hepatocyte GLUT8 deletion is sufficient to enhance peripheral caloric expenditure, attenuate high-fructose diet–induced peripheral fat accumulation, and ameliorate hepatocellular damage, steatosis, and inflammatory marker gene expression in multiple models of progressive metabolic dysfunction–associated steatotic liver disease (MASLD) and MASH. We complement genetic data by identifying and characterizing a novel small-molecule GLUT8-selective inhibitor, P20, to show that pharmacological GLUT8 blockade restores hepatocyte FGF21 and blocks fructose-induced triglyceride accumulation in vitro. We conclude that isoform-selective glucose transport blockade is a feasible and effective means to abate MASLD and MASH, at least in part by preventing excess TCA flux without provoking compensatory glutamine utilization.

## METHODS

### Mouse models and treatment

All research was conducted in accordance with both the Declarations of Helsinki and Istanbul.

All animal procedures were approved by the Washington University School of Medicine Animal Studies Committee (Approval #24055). Male GLUT8^WT^ and GLUT8^LKO^ mice were obtained by crossing GLUT8^fl/fll^ mice with transgenic mice expressing Cre recombinase under albumin promoter control, as described.^7^^,^^30^ All mice were 6–8-week-old at enrollment. High-fructose diet (TD86489) was 60% kCal content, obtained from Envigo as we reported.^2^^,^^6^^,^^16^^,^^17^ Western diet (Envigo, #TD88137) was administered with low-dose weekly carbon tetrachloride (Sigma #319961) in corn oil exactly as reported.^26^ Vehicle-treated mice were treated with corn oil alone. Finally, in 1 set of studies, mice were fed a 4-week methionine and choline-deficient diet (TD.90262, >40% sucrose, 10% corn oil). All procedures were performed in accordance with the approved guidelines by the Animal Studies Committee at Washington University School of Medicine. Primary murine hepatocytes were isolated and cultured as reported.^16^^,^^17^

### Serum analyses

Fasting blood glucose was measured via glucometer using tail vein blood. For all other serum analyses, submandibular blood collection was performed immediately before sacrifice and serum was separated. Triglycerides (Thermo Fisher Scientific #TR22421), cholesterol (Thermo Fisher Scientific #TR13421), and free fatty acids (Wako Diagnostics #999-34691, #995-34791, #991-34891, #993-35191) quantification were performed using commercially available reagents according to the manufacturer’s directions. ALT and albumin levels were quantified using an AMS LIASYS Chemistry Analyzer.

### Hepatic lipids

Lipids were extracted from ~100 mg hepatic tissue homogenized in 2:1 chloroform:methanol. Each extract (0.25%–0.5%) was evaporated for at least 1 hour before biochemical quantification of triglycerides, cholesterol, and free fatty acids using reagents described in section Serum Analyses precisely according to the manufacturer’s directions.

### Hematoxylin and eosin, picosirius red, and F4/80+ histology

Formalin-fixed 5 μm sections from GLUT8^WT^ and GLUT8^LKO^ mice were stained according to the described protocols.^6^^,^^7^

### Body composition analysis

Body composition analysis was carried out in unanesthetized mice as described^5^^,^^6^^,^^31^ using an EchoMRI 3-1 device (Echo Medical Systems) via the Washington University Diabetic Mouse Models Phenotyping Core Facility.

### Indirect calorimetry

Oxygen consumption, CO_2_ production, respiratory exchange ratio, and heat production were measured using the Phenomaster system (TSE) via the Washington University Diabetic Mouse Models Phenotyping Core Facility as described.^5^^,^^31^ Metabolic parameters were documented every 10–13 minutes.

### RNA sequencing

RNA-sequencing (RNAseq) was performed by the Washington University Genome Technology Access Center (GTAC), as we have reported.^6^^,^^7^^,^^21^ Library preparation was performed with 10 μG of total RNA with a Bioanalyzer RIN score >8.0. Ribosomal RNA was removed by poly-A selection using Oligo-dT beads (mRNA Direct kit, Life Technologies). mRNA was then fragmented in buffer containing 40 mM Tris acetate pH 8.2, 100 mM potassium acetate, and 30 mM magnesium acetate and heated to 94 degrees for 150 seconds. mRNA was reverse-transcribed to yield cDNA using SuperScript III RT enzyme (Life Technologies, per manufacturer’s instructions) and random hexamers. A second strand reaction was performed to yield ds-cDNA. cDNA was blunt-ended, had an A base added to the 3′ ends, and then had Illumina sequencing adapters ligated to the ends. Ligated fragments were then amplified for 12 cycles using primers incorporating unique index tags. Fragments were sequenced on an Illumina HiSeq-3000 using single reads extending 50 bases.

RNAseq reads were aligned to the Ensembl release 76 top-level assembly with STAR version 2.0.4b. Gene counts were derived from the number of uniquely aligned unambiguous reads by Subread:featureCount version 1.4.5. Transcript counts were produced by Sailfish version 0.6.3. Sequencing performance was assessed for the total number of aligned reads, the total number of uniquely aligned reads, genes and transcripts detected, ribosomal fraction known junction saturation, and read distribution over known gene models with RSeQC version 2.3.

To enhance the biological interpretation of the large set of transcripts, grouping of genes/transcripts based on functional similarity was achieved using the R/Bioconductor packages GAGE and Pathview. GAGE and Pathview were also used to generate pathway maps on known signaling and metabolism pathways curated by KEGG.

### Quantitative real-time RT-PCR

Quantitative real-time RT-PCR (qRT-PCR) was performed as previously reported^6^^,^^7^ with some modifications. Snap-frozen livers or cultured hepatocytes were homogenized in Trizol reagent (Invitrogen #15596026). RNA isolated according to the manufacturer’s protocol was reverse-transcribed using the Quantitect Qiagen reverse transcriptase kit (Qiagen #205310). cDNA was subjected to quantitative PCR using the SYBR Green master mix reagent (Applied Biosystems #4309155). Primers used are listed in Table 1.

**TABLE 1:** Primers used for qRT-PCR

Gene target	Forward (5′–3′)	Reverse (5′–3′)
*Acc1*	TGT CCG CAC TGA CTG TAA CCA	TGC TCC GCA CAG ATT CTT CA
*Col1a1*	GCT CCT CCT AGG GGC CAC T	CCA CGT CTC ACC ATT GGG G
*Col3a1*	CTG TAA CAT GGA AAC TGG GGA AA	CCA TAG CTG AAC TGA AAA CAA CC
*Cxcl2*	CCA ACC ACC AGG CTA CAG G	GCG TCA CAC TCA AGC TCT G
*Elovl6*	GAA AAG CAG TTC AAC GAG AAC G	AGA TGC CGA CCA CCA AAG ATA
*Fasn*	CCT GGA TAG CAT TCC GAA CCT	AGC ACA TCT CGA AGG CTA CAC A
*Fgf21*	CTG CTG GGG GTC TAC CAA G	CTG CGC CTA CCA CTG TTC C
*Gpat*	CAA CAC CAT CCC CGA CAT C	GTG ACC TTC GAT TAT GCG ATC A
*Lpk*	CTT GCT CTA CCG TGA GCC TC	ACC ACA ATC ACC AGA TCA CC
*Il1b*	GCA ACT GTT CCT GAA CTC AAC T	ATC TTT TGG GGT CCG TCA ACT
*Il6*	CTG CAA GAG ACT TCC ATC CAG	AGT GGT AT GAC AGG TCT GTT GG
*Pgc1α*	ACA CCG CAA TTC TCC CTT GT	CGG CGC TCT TCA ATT GCT TT
*Ppara*	ACG ATG CTG TCC TCC TTG ATG	GTG TGA TAA AGC CAT TGC CGT
*Scd1*	CCG GAG ACC CTT AGA TCG A	TAG CCT GTA AAA GAT TTC TGC AAA CC
*Slc2a8*	TTC ATG GCC TTT CTA GTG ACC	GAG TCC TGC CTT TAG TCT CAG
*Tgfb*	CCT GGC CCT GCT GAA CTT G	TTG ATG TGG CCG AAG TCC AAC
*Tnfa*	CAG GCG GTG CCT ATG TCT C	CGA TCA CCC CGA AGT TCA GTA G

### Radiolabeled carbohydrate uptake and stable-isotope tracing experiments

Radiolabeled 2DG and fructose uptake experiments were performed in HEK293 cells expressing GLUT1-4, GLUT5, and GLUT8, as we reported and validated previously.^27^ GLUT1-directed shRNA was stably expressed in this cell line to knock down endogenous GLUT1. The purpose of this is to minimize its contribution to uptake. Stable-isotope tracing experiments were performed precisely as described.^28^^,^^29^

### Statistics

Data were analyzed using GraphPad Prism version 9.0 (RRID:SCR_015807). *p*<0.05 was defined as statistically significant. Data shown are as mean ± SEM. Two-tailed *t* tests are used with Bonferroni-Dunn post hoc correction, where multiple comparisons are made unless otherwise noted in the figure legends.

## RESULTS

### Transcriptomic analysis reveals lower inflammatory marker gene expression in livers from whole-body GLUT8-deficient mice

**FIGURE 1:**
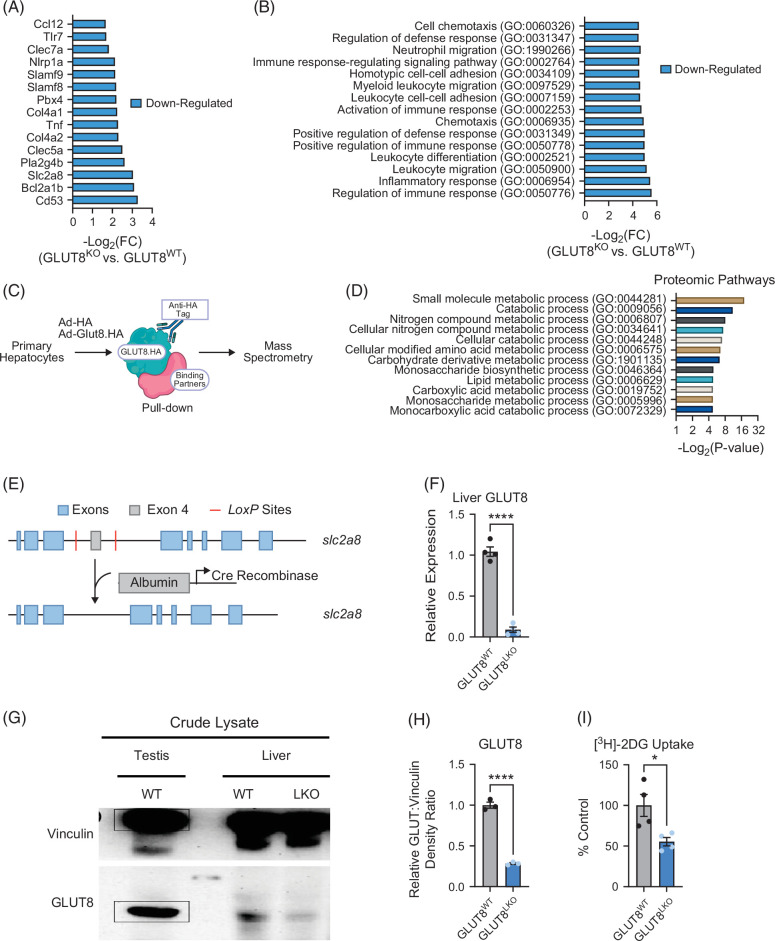
Transcriptomic analysis revealed that whole-body GLUT8 deficiency lowers inflammatory marker gene expressions while hepatocyte-specific GLUT8 deletion impairs hepatocyte glucose uptake. (A) Significantly downregulated genes identified by next-generation RNA sequencing in primary hepatocytes from whole-body GLUT8 WT and GLUT8 KO mouse liver. Adjusted *p*<0.05 in GLUT8 KO versus GLUT8 WT livers. (B) Significantly downregulated GO pathways identified by RNAseq using a threshold adjusted *p*<0.05 in GLUT8 KO versus GLUT8 WT livers. (C) Illustration of an immunoprecipitation pull-down experiment targeting HA-tagged GLUT8 to identify binding partners of GLUT8. (D) GO pathways represented by GLUT8 binding partners identified by immunoproteomic analysis using HA-tagged GLUT8 as bait. (E) Floxed *Slc2a8* construct showing *LoxP* sites flanking exon 4. Crossing with Alb-Cre transgenic mice results in exon 4 deletion and a premature stop. (F–H) qRT-PCR and immunoblot analyses quantifying GLUT8 gene expression and protein in GLUT8^WT^ and GLUT8^LKO^ mice. (I) Radiolabeled [^3^H]-2-deoxy-D-glucose uptake in isolated hepatocytes from GLUT8^WT^ and GLUT8^LKO^ mice. *, and **** represent *p*<0.05, and *p*<0.0001, respectively, by 2-tailed homoscedastic *t* test. Abbreviations: GO, Gene Ontology; qRT-PCR, quantitative real-time RT-PCR.

Germline, whole-body GLUT8-deficient mice, and mice treated with GLUT8 gene-silencing oligonucleotides exhibit increased calorie expenditure, increased hepatocyte fasting-like signaling through the PPARα pathway, and protection from hepatic steatosis in diet-induced obese models.^7^^,^^13^^,^^14^^,^^16^^–^^18^ We first defined hepatocyte gene-specific and transcriptional perturbations in isolated cultured primary murine hepatocytes derived from whole-body GLUT8-deficient liver (Figure 1A). This revealed lower basal inflammatory and fibrogenic gene expression, including Ccl12, TNF, Col4a1/Col4a2, and TLR7. In addition, among the most downregulated factors in GLUT8KO hepatocytes was the B-cell development factor and tetraspanin, CD53 (Figure 1A). Validating this result functionally, we recently reported that germline deletion of CD53 was sufficient to attenuate diet-induced liver fat accumulation and inflammatory signaling in hepatocytes.^30^ Gene Ontology pathway analysis of significantly downregulated genes revealed inhibition of multiple inflammatory pathways in isolated wild-type (WT) and GLUT8-deficient (GLUT8KO) hepatocytes cultured in nutrient-replete (eg, regular growth media) conditions (Figure 1B). Whole-body GLUT8 deletion significantly inhibited hepatocyte gene pathways, including cell chemotaxis and adhesion, defense and immune responses, inflammatory responses, and leukocyte differentiation (Figure 1B). We next took an orthogonal, proteomics-based approach to agnostically frame broader GLUT8 function (Figure 1C). We overexpressed either GFP alone or HA-tagged GLUT8 in primary hepatocytes, followed by anti-HA immunoprecipitation and protein mass spectroscopy. This revealed binding partners enriched for carbohydrate, nitrogenous, and other small molecular compound metabolism. Overall, the data indicate that GLUT8 has hepatocyte-intrinsic metabolic and inflammatory functions.

### Hepatocyte-specific GLUT8 deletion impairs hepatocyte glucose uptake

These data prompted us to generate hepatocyte-specific GLUT8-deficient mice by inserting *loxP* sites flanking exon 4 of *slc2a8*, the gene that encodes GLUT8 (Figure 1E). We crossed mice homozygous for these *loxP* insertions with hemizygous male transgenic mice expressing Cre recombinase under hepatocyte-specific albumin promoter control. Cre-mediated excision in these mice results in splicing exons 3 and 5, which induces a frameshift mutation and premature stop codon. GLUT8^LKO^ mice are live born, morphologically normal, and reproduce in Mendelian ratios. qRT-PCR analysis of crude liver mRNA confirmed >95% reduction in liver GLUT8 gene expression (Figure 1F). We confirmed a reduction in GLUT8 protein abundance by immunoblot analysis in crude liver protein extracts using mouse testis as a positive band control (Figures 1G, H). To functionally validate mRNA and protein reductions in *slc2a8*/GLUT8, we showed that hepatocyte-specific GLUT8 deletion yields a ~50% reduction in radiolabeled [^3^H]-2-deoxy-D-glucose uptake (Figure 1I).

### Hepatocyte GLUT8 deletion blocks carbohydrate flux through the TCA cycle without provoking glutamine anaplerosis

**FIGURE 2:**
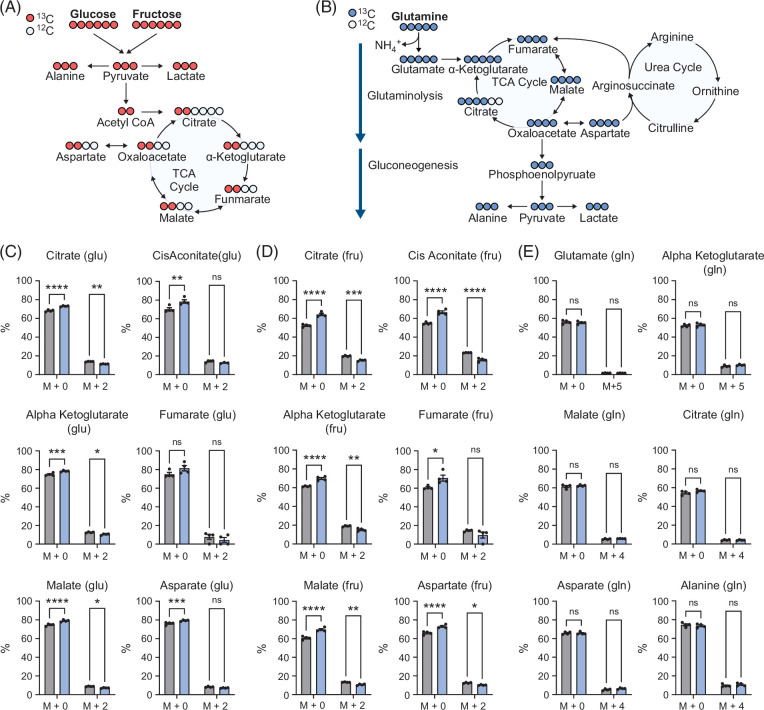
Stable isotope tracing reveals impaired glucose and fructose flux through the TCA cycle without glutamine anaplerosis. (A, B) Schematic of metabolic labeling of TCA cycle intermediaries using glucose and fructose (A), and glutamine (B) tracers. (C, D) % labeling by universally labeled [^13^C]-glucose and [^13^C]-fructose tracer into citrate, cis-aconitate, alpha-ketoglutarate, fumarate, malate, and aspartate. (E) % labeling by [^13^C]-glutamine to glutamate, alpha-ketoglutarate, malate, citrate, aspartate, and alanine. *, **, ***, and **** represent *p*<0.05, *p*<0.01, *p*<0.001, and *p*<0.0001, respectively, by 2-way ANOVA with Sidak’s post hoc correction for multiple comparisons.

Excess glutamine flux is a hallmark of MASH,^24^ which pushes excess nitrogen through an already impaired urea cycle.^31^^,^^32^ One barrier to nonselective GLUT inhibition is that preventing carbohydrate flux through the TCA cycle is compensated by glutamine anaplerosis.^25^ We therefore defined the metabolic consequences of GLUT8-specific deletion in hepatocytes through detailed stable-isotope glucose, fructose (Figure 2A), and glutamine (Figure 2B) tracing in isolated GLUT8^WT^ and GLUT8^LKO^ hepatocytes in complete (nutrient-replete) growth media. Substrate labeling revealed impaired labeled citrate (M + 2), alpha-ketoglutarate (M + 2), and malate (M + 2) with concomitant increased unlabeled glucose incorporation into the TCA cycle in GLUT8^LKO^ versus GLUT8^WT^ hepatocytes (Figure 2C). Impaired labeled fructose incorporation was even more pronounced, as GLUT8^LKO^ mice exhibited impaired citrate (M + 2), cis-aconitate (M + 2), alpha-ketoglutarate (M + 2), malate (M + 2), and aspartate (M + 2) and compensatory increased unlabeled substrate incorporation (Figure 2D). In contrast, glutamine (M + 5) labeling through the TCA cycle surprisingly indicated no significant changes in TCA cycle flux, in aspartate or alanine labeling (Figure 2E). Together, the data show that hepatocyte GLUT8-deletion impairs carbohydrate flux without provoking glutamine anaplerosis.

### Increased energy expenditure and resistance to high-fructose diet–induced peripheral adiposity in GLUT8^LKO^ mice

**FIGURE 3:**
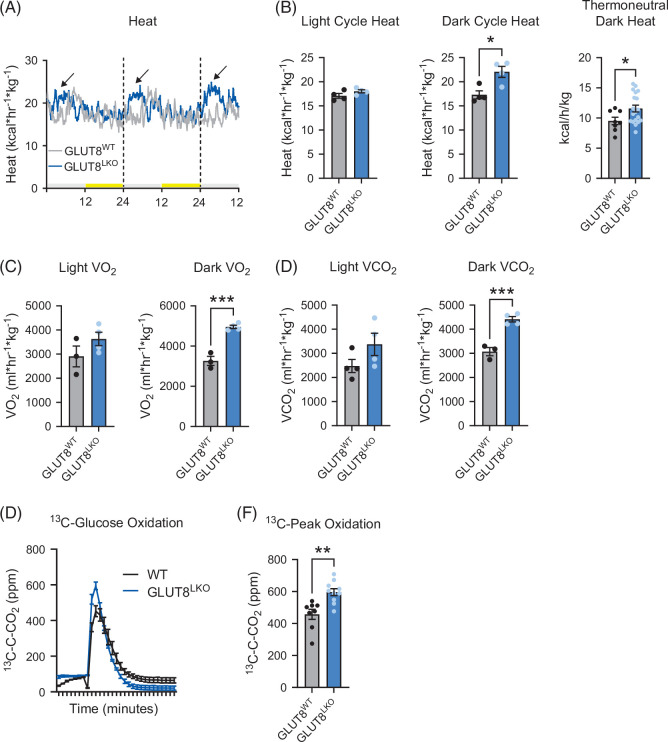
Enhanced dark-cycle energy expenditure in chow-fed liver-specific GLUT8-deficient (GLUT8^LKO^) mice. (A) Heat production versus time in GLUT8^LKO^ versus GLUT8^WT^ mice over 2.5 circadian cycles. Gray and yellow bars denote light and dark cycles. Arrows point to the enhancement of heat generation at the beginning of each dark phase. (B) Quantification of mean light cycle (ZT 0–12, left) and dark cycle (ZT 12–24, mid) heat generation at ambient and thermoneutrality (ZT 12–24, right). (C, D) Quantification of mean light and dark cycle VO_2_ (C) and VCO_2_ (D). (E, F) Whole-body glucose oxidation of ^13^C-glucose by quantification of exhaled ^13^CO_2_ graphed over time (E) and quantified at peak height (F). *, **, and *** represent *p*<0.05, *p*<0.01, and *p*<0.001, respectively, by 2-tailed homoscedastic *t* test.

**FIGURE 4:**
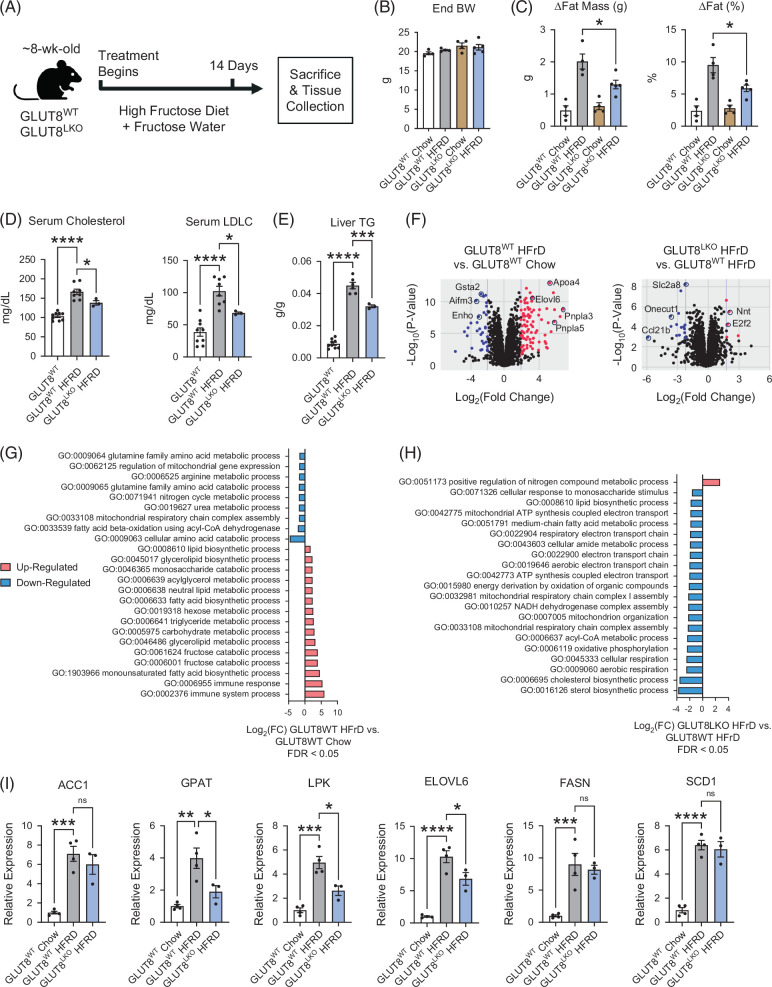
Resistance to fructose-induced peripheral fat accumulation in GLUT8^LKO^ mice. (A) Experimental design showing exposure to a high-fructose diet and high-fructose water (14 d) in GLUT8^LKO^ and GLUT8^WT^ mice. (B) Endpoint body weight in GLUT8^LKO^ versus GLUT8^WT^ mice on 14-day chow and HFrD protocol. (C) EchoMRI data showing change in body fat mass from baseline in GLUT8^LKO^ versus GLUT8^WT^ mice on 14-day chow and HFrD. (D, E) Serum lipid (D) and liver TG (E) quantification demonstrating lower LDL-C and hepatic TG in HFrD-fed GLUT8^LKO^ versus GLUT8^WT^ mice. (F–H) Volcano plot of significantly altered genes (F), and significantly altered gene ontology pathways (G, H) and in HFrD-fed GLUT^WT^ versus GLUT8^LKO^ livers (left) and in GLUT8^LKO^ HFrD- versus GLUT8^WT^ HFrD-fed livers (right). (I) qRT-PCR data quantifying de novo lipogenic gene expression. *, **, ***, and **** represent *p*<0.05, *p*<0.01, *p*<0.001, and *p*<0.0001, respectively, by 1-way ANOVA with Dunnett’s post hoc correction (panels D, E, and I), or 2-way ANOVA with Sidak’s post hoc correction (panel C).

We previously showed enhanced whole-body oxidative metabolism in germline, whole-body GLUT8^KO^ mice^7^^,^^17^ and in mice treated with the GLUT inhibitor, trehalose.^5^^,^^19^ To define if hepatocyte-specific GLUT8 deficiency is sufficient to increase calorie expenditure, we quantified basal energy expenditure in 8-week-old, chow-fed male GLUT8^WT^ and GLUT8^LKO^ mice. This revealed a consistent, rapid increase in heat generation during each dark cycle (eg, ZT 12–24) at ambient room temperature and at thermoneutrality (Figures 3A, B). This was associated with selectively increased VO_2_ and VCO_2_ during the dark cycle (Figures 3C, D), and with greater peak labeled whole-body [^13^C]-U-glucose oxidation in GLUT8^LKO^ versus GLUT8^WT^ (Figures 3E, F). Next, in an in vivo diet-induced simple steatosis model, we subjected 8-week-old GLUT8^WT^ and GLUT8^LKO^ males to a 14-day high-fructose diet (HFrD, 60% energy kcal/d, 30% w/v fructose drinking water, Figure 4A). Endpoint body weight in chow- and HFrD-fed GLUT8^WT^ and GLUT8^LKO^ was not significantly different (Figure 4B). However, HFrD-fed GLUT8^LKO^ mice gained significantly less body fat mass and proportionally less body fat mass when compared with HFrD-fed GLUT8^WT^ mice (Figure 4C). Serum lipid analysis revealed selectively increased serum LDL-C and hepatic triglyceride content in GLUT8^WT^ mice, whereas GLUT8^LKO^ mice were protected from both (Figures 4D, E). This is associated with lower induction of lipid biosynthetic gene expression by global transcriptomic analysis (Figures 4F–H) in livers from HFrD-fed GLUT8^LKO^ mice, versus GLUT8^WT^ mice. We further evaluated the reduction of genes in lipid biosynthesis in (eg, GPAT, LPK, and ELOVL6) by qRT-PCR in crude liver from HFrD-fed GLUT8^LKO^ mice, versus GLUT8^WT^ mice (Figure 4I).

### GLUT8 mediates fatty acid- and LPS-induced fat accumulation and inflammatory gene expression

**FIGURE 5:**
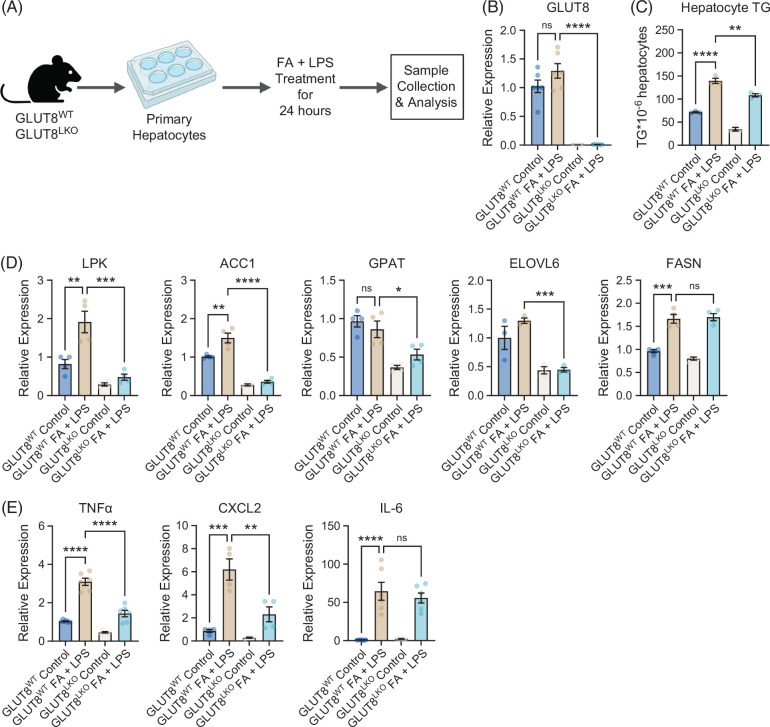
GLUT8 mediates fatty acid- and LPS-induced fat accumulation and inflammatory marker gene expression in isolated hepatocytes. (A) Experimental diagram of primary murine hepatocyte isolation, culture, and treatment with BSA-conjugated fatty acids and lipopolysaccharide (FA + LPS, 24 h) before endpoint analyses. (B) qRT-PCR data showing GLUT8 gene expression in isolated hepatocytes from GLUT8^LKO^ and GLUT8^WT^ mice treated with or without FA + LPS. (C) Enzymatic-colorimetric triglyceride quantification in isolated GLUT8^LKO^ and GLUT8^WT^ hepatocytes treated with or without FA + LPS. (D, E) qRT-PCR quantification of genes mediating de novo lipogenesis (D) and inflammation (E) in isolated GLUT8^LKO^ and GLUT8^WT^ primary hepatocytes treated with or without FA+ LPS. *, **, ***, and **** represent *p*<0.05,<0.01,<0.001, and *p*<0.0001, respectively, by 2-way ANOVA with Sidak’s post hoc test.

Inflammatory gene expression markers were reduced in isolated primary murine hepatocytes from germline whole-body GLUT^KO^ mice (Figure 1). We previously reported that hepatocytes exposed to nonessential fatty acids with low-dose lipopolysaccharide upregulate triglyceride accumulation and inflammatory gene expression.^19^^,^^30^^,^^33^ We therefore tested if GLUT8^LKO^ hepatocytes cell-intrinsically resist metabolic inflammation using this in vitro model (Figure 5A). We exposed isolated primary hepatocytes from GLUT8^WT^ and GLUT8^LKO^ male mice to 500 μM 1:1:1:1 BSA-conjugated palmitate:oleate:stearate:linoleate (24 h) with 50 ng/mL LPS (Hereafter, FA + LPS). We first confirmed nearly undetectable GLUT8 expression in isolated GLUT8^LKO^ hepatocytes (Figure 5B). Moreover, FA + LPS increased hepatocyte TG accumulation in FA + LPS-treated GLUT8^WT^ hepatocytes, and this was attenuated in GLUT8^LKO^ hepatocytes (Figure 5C). Accordingly, de novo lipogenic gene expression of LPK, ACC1, and GPAT, but not FASN, was reduced in FA + LPS-treated GLUT8^LKO^ hepatocytes when compared with GLUT8^WT^ hepatocyte controls (Figure 5D). In addition, inflammatory gene markers TNFα and CXCL2 were reduced in FA + LPS-treated GLUT8^LKO^ hepatocytes when compared with GLUT8^WT^ hepatocytes (Figure 5E). We conclude that hepatocyte GLUT8 deletion cell-intrinsically reduces the hepatocyte response to metabolic inflammatory stimuli.

### GLUT8 mediates dyslipidemia and hepatic steatosis in a murine model of MASH

**FIGURE 6:**
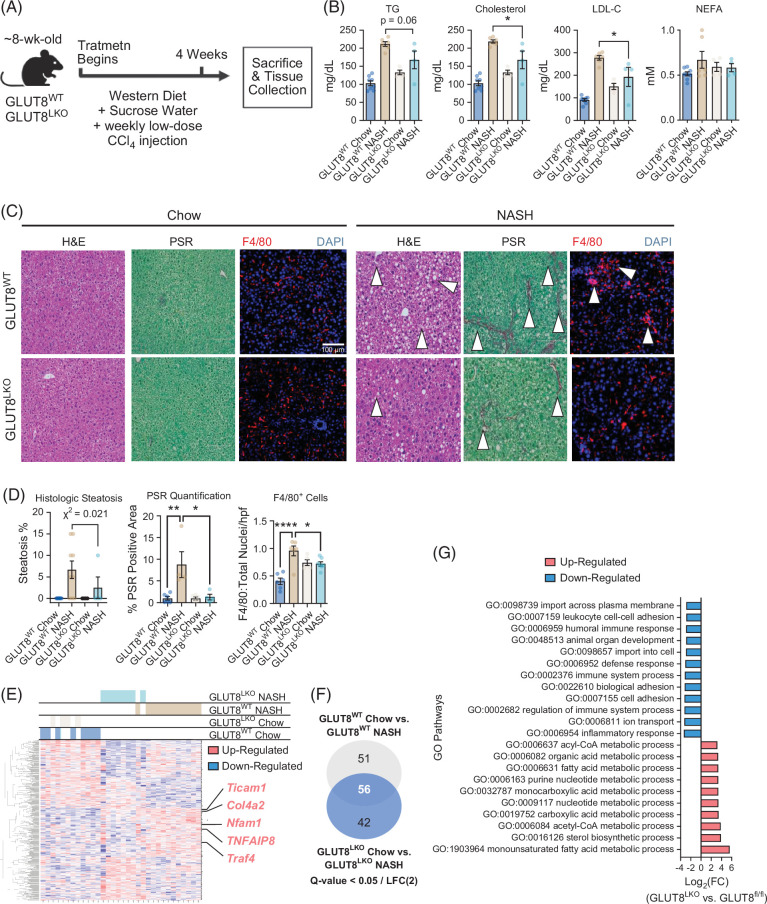
GLUT8 mediates dyslipidemia and hepatic steatosis in a chronic Western Diet and low-dose CCl_4_ model of MASH in vivo. (A) Experimental design demonstrating enrollment of germline GLUT^WT^ and GLUT8^LKO^ diet, sucrose water, and CCL_4_ exposure (MASH diet, 4 wk) before sacrifice and analysis. (B) Enzymatic-colorimetric serum lipid quantification in GLUT8^LKO^ and GLUT8^WT^ mice fed chow or MASH diet. (C) H&E, PSR, and F4/80 staining of livers from GLUT8^LKO^ and GLUT8^WT^ livers after 4-week chow or MASH diet treatment. (D) Blinded pathologist scoring of steatosis, quantification of PSR, and F4/80^+^ cells in GLUT8^LKO^ and GLUT8^WT^ liver micrographs after 4-week chow or MASH diet treatment. (E) Unsupervised clustering and heat map demonstrating gene expression comparing bulk transcriptomes from GLUT8^LKO^ and GLUT8^WT^ livers after chow and MASH diet exposure. (F) Significantly altered genes (*Q*<0.05, Log(FC) >2) in GLUT8^LKO^ chow versus MASH, in GLUT8^WT^ chow versus MASH, and the overlap in significantly altered genes in each set. (G) Pathway analyses demonstrating the most highly downregulated and upregulated GO pathways when comparing livers from GLUT8^LKO^ and GLUT8^WT^ mice fed the MASH diet. *, **, and **** represent *p*<0.05, *p*<0.01, *p*<0.0001, and *p*<0.0001, respectively, by 2-way ANOVA with Sidak’s post hoc test or by chi-squared analysis. Abbreviations: GO, Gene Ontology; H&E, hematoxylin and eosin; PSR, picosirius red.

The data prompted us to examine in vivo metabolic inflammatory responses in GLUT8^WT^ and GLUT8^LKO^ mice. We placed germline GLUT8^WT^ and GLUT8^LKO^ mice on a 28-day MASH diet (Figure 6A). Serum TG, total cholesterol, and LDL-C were significantly elevated in MASH diet-fed GLUT8^WT^ mice (Figure 6B). Cholesterol and LDL-C in GLUT8^LKO^ mice were significantly lower, with a trend toward lower serum TG when compared with GLUT8^WT^ mice (Figure 6B). We confirmed these biochemical measurements of protection by histologic analysis of sections from GLUT8^WT^ and GLUT8^LKO^ livers from mice fed chow or NASH diets (Figure 6C). Hematoxylin and eosin staining, picosirius red staining, and F4/80 immunofluorescence revealed increased steatosis, collagen staining, and F4/80^+^ cellular infiltrate in the GLUT8^LKO^ mouse liver when compared with GLUT8^WT^ mice. This was confirmed by treatment- and genotype-blinded histopathologic scoring, which also revealed a significant reduction in the proportion of mice at or above the case definition threshold for histologic steatosis (defined as ≥5% steatosis), picosirius red–stained area, and F4/80^+^ cells in MASH-fed GLUT8^LKO^ mice when compared with GLUT8^WT^ controls (Figure 6D). Livers from chow- and MASH-fed GLUT8^WT^ and GLUT8^LKO^ mice were subjected to bulk transcriptomics and unsupervised clustering analysis. Unsupervised clustering demonstrated overlapping, interspersed transcriptomic profiles when comparing chow-fed GLUT8^WT^ and GLUT8^LKO^ livers (Figure 6E, leftward clusters). In contrast, MASH diet exposure resulted in separation in hepatic transcriptomic clustering in GLUT8^WT^ and GLUT8^LKO^ livers (Figure 6E, rightward clusters). Moreover, MASH-fed GLUT8^LKO^ liver transcriptomes clustered more closely with both chow-fed transcriptomes in the middle of the unsupervised clustering than did the MASH-fed GLUT8^WT^ liver transcriptomes (Figure 6E). In total, 107 genes met a stringent significance threshold of *Q*<0.05 and LFC >2 in MASH-fed versus chow-fed GLUT8^WT^ livers. Ninety-eight genes met the same significance threshold in MASH-fed versus chow-fed GLUT8^LKO^ livers. Fifty-six of these genes were shared as significantly regulated in both genotypes. GLUT8^LKO^ mice also significantly altered 42 genes apart from those in the MASH-fed WT state (Figure 6F). Specific genes of interest that were significantly lower in GLUT^LKO^ versus GLUT^WT^ liver included inflammatory signaling genes (ticam1 and nfam1), TNFα-related genes (tnfaip8 and traf4), and fibrosis-related genes (col4a1, Figure 6E). Gene Ontology pathway analysis revealed the most profoundly downregulated processes (GLUT8^LKO^ MASH vs. GLUT8^WT^ MASH) included immune response activation, inflammatory responses, leukocyte cell-cell adhesion, defense responses, and immune regulation (Figure 6G). This was corroborated in downregulated KEGG pathways, which included IL-17, Rap1 signaling, chemokine signaling, NFKB, and TNFα signaling (Supplemental Figure S2, http://links.lww.com/HC9/C128). In contrast, processes upregulated in GLUT8^LKO^ relative to GLUT8^WT^ liver included Gene Ontology pathways related to metabolism of acyl-CoA, organic acids, fatty acids, acetyl-CoA, and monounsaturated fatty acids, and KEGG pathways related to fatty acid and branched chain amino acid catabolism and oxidative phosphorylation (Figure 6G and Supplemental Figure S2, http://links.lww.com/HC9/C128). Together, in vitro modeling and in vivo data support the conclusion that hepatocyte GLUT8 mediates metabolic inflammation.

**FIGURE 7:**
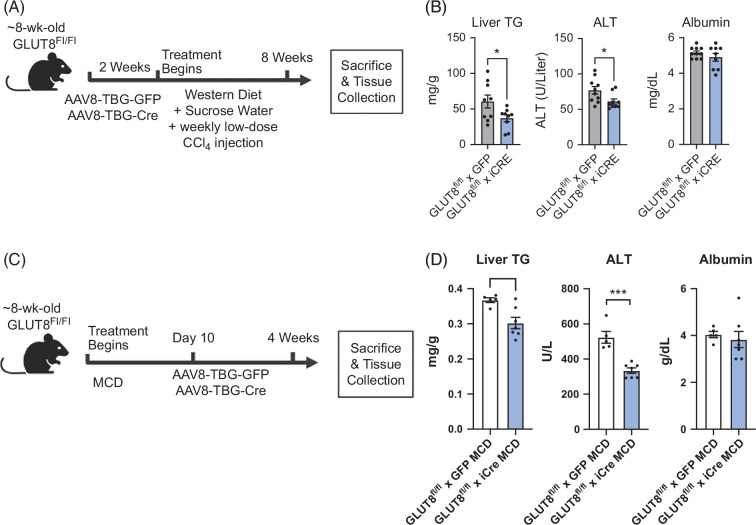
Hepatoprotective effect of hepatocyte GLUT8 deletion extends to intervention models and acute models of MASH and mediates hepatic TG accumulation and hepatocellular damage. (A) Experimental setup demonstrating inducible GLUT8 deletion 2 weeks before 56-day Western Diet and low-dose CCl_4_ exposure before inducible hepatocyte-specific GLUT8 deletion before sacrifice and analysis. (B) Liver TG, serum ALT and serum albumin in GLUT8^WT^ and GLUT8^LKO^ mice exposed to a 56-day Western Diet and low-dose CCl_4_. (C) Experimental setup demonstrating MCD diet exposure before inducible hepatocyte-specific GLUT8 deletion before sacrifice and analysis. (D) Liver TG, serum ALT and serum albumin in GLUT8^WT^ and GLUT8^LKO^ mice exposed to a 28-day MCD. *, ***, and **** represent *p*<0.05,<0.001, and<0.0001, respectively, by 2-tailed homoscedastic *t* test. Abbreviation: MCD, methionine- and choline-deficient diet.

### Hepatoprotective effects of hepatocyte GLUT8 deletion extend across distinct chronic and intervention models of MASH

We turned toward distinct in vivo MASH models to test the hypothesis that hepatocyte GLUT8 targeting exerts a generalizable hepatoprotective effect. We first tested if acute hepatocyte GLUT8 deletion reduced hepatic TG and transaminase elevation using the MASH diet model (Figure 7A). We treated GLUT8^fl/fl^ mice with AAV8 encoding TBG promoter-driven-GFP or -iCre (eg, AAV8-TBG-GFP or AAV8-TBG-iCre), then exposed mice to a 6-week MASH diet (eg, Western Diet, sucrose drinking water, and low-dose weekly carbon tetrachloride, Figure 7A).^26^ Again, MASH diet–exposed GLUT8^fl/fl^ × iCre mice had significantly lower endpoint liver TG and circulating serum ALT, a marker of hepatocellular damage, with no reduction in serum albumin, a marker of hepatocyte synthetic function (Figure 7B). We then examined a disease intervention model to assess the effects of GLUT8 deletion after the onset of dietary insult. Eight-week-old male GLUT8^fl/fl^ mice were treated with a methionine and choline-deficient diet 10 days before treatment with AAV8-TBG-GFP or -iCre before sacrifice and analysis after 28 days of methionine and choline-deficient diet exposure (Figure 7C). Again, GLUT8^fl/fl^ × iCre mice had significantly lower endpoint liver TG and ALT without reductions in serum albumin, when compared with GLUT8^fl/fl^ × GFP mice (Figure 7D). These data indicate a generalizable protective effect of GLUT8 blockade independent of the timing of GLUT8 deletion or dietary insult.

### Selective pharmacological GLUT8 blockade reverses hepatocyte TG accumulation and inflammatory marker gene expression

**FIGURE 8:**
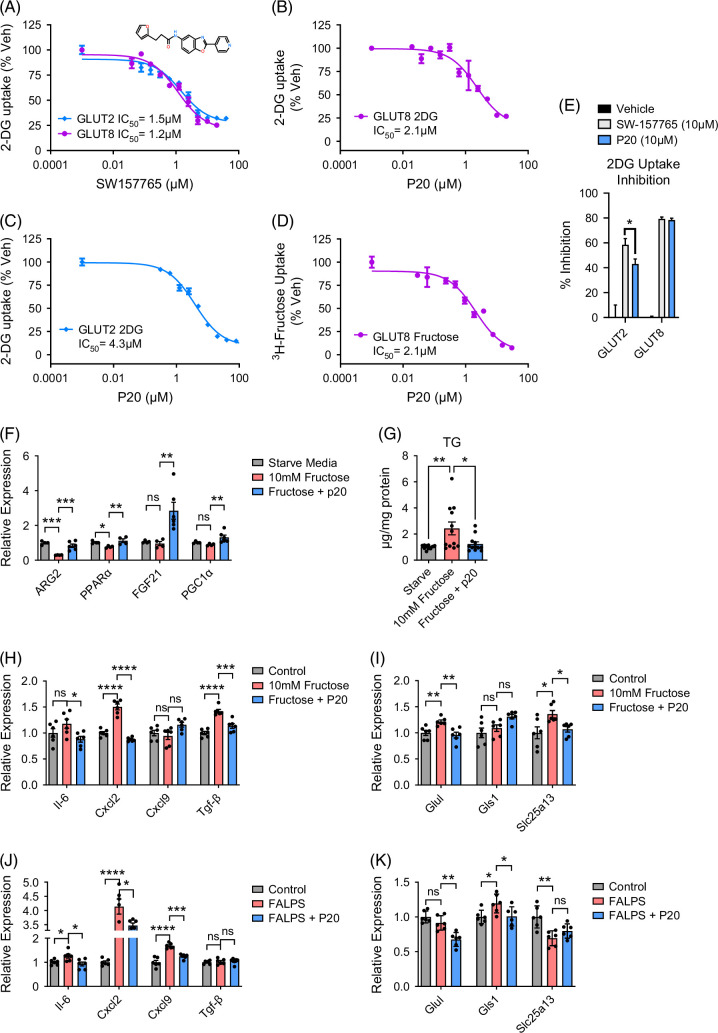
Selective pharmacological GLUT8 blockade reverses fructose-mediated fasting gene suppression and TG accumulation. (A) IC_50_ analysis for compound SW-157765 against GLUT2 and GLUT8-mediated [^3^H]-2-deoxyglucose uptake. (B, C) IC_50_ for compound P20 against GLUT8 (B) and GLUT2-mediated (C) [^3^H]-2-deoxyglucose uptake. (D) IC_50_ for compound P20 against GLUT8-mediated fructose uptake. (E) Relative GLUT2 and GLUT8 inhibition by SW-157765 and P20. (F) qRT-PCR analysis of fasting-induced hepatic gene expression in response to excess fructose with or without P20 treatment. (G) Enzymatic-colorimetric triglyceride quantification in isolated GLUT8^LKO^ and GLUT8^WT^ hepatocytes treated with or without excess fructose exposure. (H, I) qRT-PCR analysis of inflammatory (H) and glutamine metabolism (I) gene expressions in response to excess fructose with or without P20 treatment in hepatocytes in vitro. (J, K) qRT-PCR analysis of inflammatory (J) and glutamine metabolism (K) gene expression in response to FA and LPS with or without P20 treatment in hepatocytes in vitro. *, **, ***, and **** represent *p*<0.05,<0.01,<0.001, and *p*<0.0001, respectively, by 1-way ANOVA with post hoc correction.

We set out to identify isoform-selective small-molecule GLUT8 inhibitors and then test their in vitro efficacy against fructose-induced TG accumulation. We first tested compound SW-157765, which was first identified as a GLUT8 inhibitor in a 200,000-compound chemical screen for drugs that are lethal to non–small-cell lung tumors.^11^ We quantified the ability of SW-157765 to selectively block GLUT8 and GLUT2—the 2 most highly expressed hepatocyte carbohydrate transporters.^17^ To do this, we utilized our established GLUT isoform-specific 293 cell screening platform.^2^^,^^27^ Briefly, this cell line expresses shRNA targeting its endogenous GLUT1 transporter and concomitantly overexpresses the screened GLUT isoform. For purposes here, these cells specifically express either GLUT2 or GLUT8 (Figure 8) or GLUT1, -3, -4, or -5 (Supplemental Figure S3, http://links.lww.com/HC9/C128). On these platforms, we defined substantial overlap in SW-157765 potency against both GLUT2 and GLUT8. Both isoforms were maximally inhibited at 25%–30% uptake, with an IC_50_ for GLUT2 and GLUT8 with IC_50_ of 1.5 and 1.2 μM, respectively (Figure 8A). Using the same screening platform, we identified a compound series, “P20,” from a Lilly compound library screen.^27^ P20 exhibited an IC_50_ for GLUT8 inhibition similar to that of SW-157765 (2.1 μM, Figures 8B, C), but its GLUT2 IC_50_ was 2.9-fold higher than that of SW-157765 (4.3 μM, Figure 8D). P20 also inhibited GLUT8-mediated fructose uptake with an IC_50_ of 2.1 μM. Maximal transport inhibition for P20 to block GLUT8-mediated 2DG and fructose transport each exceeded 80% (Figure 8D). Percentage inhibition comparing the 2 compounds head-to-head at 10 μM inhibitor dosage in isolated primary murine hepatocytes was significantly greater for P20 when compared with SW-157765 (Figure 8E). In addition to improved GLUT8:GLUT2 selectivity, P20 was even more selective for GLUT8 against other class I GLUTs: GLUT1, -3, and -4, as well as the GLUT5 fructose-only transporter (Supplemental Figure S3, http://links.lww.com/HC9/C128). For these transporters, the P20 IC_50_ ranged from 7.7 μM (GLUT4) to indeterminate (GLUT1, eg, no measurable inhibitory activity) at the concentrations tested (Supplemental Figure S1, http://links.lww.com/HC9/C128).

We examined the hypothesis that P20 reverses fructose-induced suppression of key fasting-responsive genes in hepatocytes. Fructose-treated (10 mM) hepatocytes suppressed Arg2 and PPARα gene expression (Figure 8F), and 10 μM P20 treatment in the presence of fructose reversed this suppression. P20 also induced fasting signaling factors fibroblast growth factor 21 (FGF21^34^^,^^35^) and PGC1α, despite the presence of 10 mM fructose, when compared with cultures treated with fructose alone. (Figure 8F). Finally, fructose induced TG accumulation in isolated wild-type murine hepatocytes, whereas P20 reversed fructose-induced hepatocyte TG accumulation (Figure 8G). Similarly, P20 lowered fructose-induced inflammatory gene markers *Il6*, *Cxcl2*, and *Tgf-β* (Figure 8H) and glutamine metabolic genes *Glul* and *Slc25a13* (Figure 8I). In addition, hepatocyte cultures exposed to FA + LPS had lower *Il6*, *Cxcl2*, *Cxcl9*, *Glul*, *Gls1*, and *Slc25a13* when compared with fructose-exposed hepatocytes treated with vehicle (Figures 8J, K). Together, these results suggest that selective pharmacological GLUT inhibition restores fasting-like hepatocyte signaling responses, and blocks triglyceride, inflammatory, and glutamine metabolic changes.

## DISCUSSION

Targeting glucose transporters has been feasible for decades. However, nonspecific GLUT inhibitors such as phloretin, phlorizin, and cytochalasin B are used primarily as tools in classical glucose transporter experiments, and not as therapeutic agents. The use of GLUT inhibitors is currently limited by the lack of isoform selectivity for these agents. GLUT isoform selectivity is critical due to the broad tissue distribution and functions of the GLUT family.

The human consequences of nonselective GLUT targeting are illustrated by first-generation HIV protease inhibitors, which induce peripheral glucose intolerance.^36^ This is further underscored by genetic and pharmacological data in animal models and human subjects, demonstrating the isoform-specific sequelae of off-target GLUT inhibition. GLUT1 deletion imparts GLUT1 deficiency syndrome,^37^ comprising seizures and choreoathetoid movement. GLUT2 blockade in the liver impairs beta cell competence and bile acid homeostasis,^8^ whereas whole-body GLUT2 deficiency yields hepatorenal glycogenosis and impaired glucose and galactose utilization (eg, Fanconi-Bickel Syndrome). Pharmacological and genetic GLUT4 blockade in humans and rodents impairs glucose and insulin tolerance in almost any compartment.^36^^,^^38^^,^^39^ Beyond the class I glucose transporters (GLUT1-4), inhibiting class II and III transporters GLUT10 and GLUT9 induce arterial tortuosity syndrome and hyperuricemia,^9^^,^^40^ respectively. These preclinical and clinical findings juxtapose with promising data that blocking SGLT^41^ and GLUT family transporters can potentially abate several disease processes, including cancer,^3^^,^^15^ autoimmune/inflammatory,^42^ and metabolic disease.^2^^,^^6^^,^^16^^,^^17^^,^^19^ Therefore, precision glucose transport targeting is a barrier to address toward GLUT-based therapies.^43^

On that basis, there are 3 principal findings in this work. First, hepatocyte-specific genetic GLUT8 inhibition is sufficient to convey the metabolic effect of whole-body GLUT8 targeting. Second, GLUT8 blockade reduces TCA cycle flux. Expectedly, this induces compensatory carbon flux through the TCA cycle, yet it does not provoke glutamine anaplerosis. Third, despite high homology, we achieved pharmacological separation between the 2 most abundant hepatocyte GLUTs—GLUT2 and GLUT8.

These new data demonstrate the efficacy of compartment- and isoform-specific GLUT8 blockade. Postdevelopmental GLUT8 targeting is promising in part because no deleterious metabolic consequences are identified in whole-body GLUT8 blockade,^7^^,^^11^^,^^16^^–^^18^ and because antisense, pharmacological, and genetic whole-body GLUT8 targeting reveal multiple avenues to target this pathway.^2^^,^^5^^,^^7^^,^^16^^–^^19^ Moreover, pharmacological inhibitors of varying potency and specificity exist to inform advanced GLUT targeting approaches.^11^^,^^30^^,^^33^^,^^44^^,^^45^ We extend these data to show that hepatocyte GLUT8 deletion is sufficient to drive whole-body energy expenditure, peripheral fat accumulation, and protect from hepatic lipid accumulation, inflammatory gene expression, and transaminase elevation in response to multiple diet-induced insults. This is particularly important because GLUT8 is abundantly expressed in the heart, testis, and brain. In addition, some reports indicate an association between whole-body GLUT8 deletion, smaller litter size without clear etiology,^13^ and alterations in behavior and locomotor activity.^23^ The data also support the assertion that GLUT8 targeting extends hepatoprotection beyond carbohydrate overloading.

Some additional barriers to address remain: (i) GLUT targeting and fasting-like metabolic pathways may be sexually dimorphic, (ii) leveraging pathways activated downstream of the GLUTs (and of fasting in general) may render even greater therapeutic specificity,^4^^,^^6^^,^^28^^,^^29^^,^^31^^,^^46^ and (iii) no compartment- or GLUT8-selective compounds are yet available. We advance this paradigm through hepatocyte-specific genetic GLUT8 deletion, and by identifying a GLUT8-selective and potent first-in-class GLUT8-selective inhibitor, P20. Importantly, the 2.9-fold selectivity index for P20 demonstrates that pharmacological GLUT isoform selectivity is feasible, despite high GLUT isoform homology.^1^ P20 reversed fructose-induced TG accumulation and fructose suppression of Arg2 and FGF21 expression in vitro. This recapitulated important glucose fasting-mimetic outcomes previously identified after treatment with the natural disaccharide GLUT inhibitor, trehalose^2^^,^^5^^,^^6^ and lactotrehalose.^19^^,^^47^ Nevertheless, the data justify subsequent rational design and in vivo testing required to further optimize this selectivity.

Finally, a forward-looking aspect of these data should also be highlighted. TCA cycle flux and anaplerosis are increased in human MASH.^24^^,^^29^^,^^48^ Consistent with this, attenuating substrate flux through the TCA cycle alleviates MASH at the level of the carbohydrate transporter, gluconeogenesis,^48^ the mitochondrial pyruvate carrier,^49^ and blockade of glutamine anaplerosis through GLS1.^50^ We show here that the metabolic stress of GLUT blockade in the hepatocyte here reduces exogenous carbon input into the TCA cycle and forces compensatory endogenous carbon fuel utilization independent of glutamine anaplerosis. In contrast, aspartate labeling from glutamine tracer shows that arginase 2 (Arg2) deletion in the mitochondrial urea cycle impairs TCA cycle flux, prompts compensatory glutamine anaplerosis, and predisposes to age-induced MASLD and MASH in mouse models, MASH-derived human organoids, and in prospective human metabolomic studies. The data are thus consistent with the provocative hypothesis that glutamine anaplersosis per se is contributory toward MASH and MASLD pathogenesis. This cause-consequence relationship remains to be more deeply interrogated. Nevertheless, the data demonstrate the translational potential of hepatocyte carbohydrate transporter blockade in multiple models of simple steatosis and MASH. We nominate GLUT8 as an exemplar target, blockade of which attenuates TCA cycle flux without invoking glutamine anaplerosis, and extend these basic findings to identify P20 as a newly recognized selective GLUT8 inhibitor to treat metabolic, malignant^3^^,^^11^^,^^15^ disease and perhaps beyond.

## Supplementary Material

## References

[1] Thorens B Mueckler M . Glucose transporters in the 21st Century. Am J Physiol Endocrinol Metab. 2010;298:E141–E145. 20009031 10.1152/ajpendo.00712.2009 PMC2822486

[2] DeBosch BJ Heitmeier MR Mayer AL Higgins CB Crowley JR Kraft TE . Trehalose inhibits solute carrier 2A (SLC2A) proteins to induce autophagy and prevent hepatic steatosis. Sci Signal. 2016;9:ra21. 26905426 10.1126/scisignal.aac5472 PMC4816640

[3] McBrayer SK Cheng JC Singhal S Krett NL Rosen ST Shanmugam M . Multiple myeloma exhibits novel dependence on GLUT4, GLUT8, and GLUT11: Implications for glucose transporter-directed therapy. Blood. 2012;119:4686–4697. 22452979 10.1182/blood-2011-09-377846 PMC3367873

[4] Higgins CB Mayer AL Zhang Y Franczyk M Ballentine S Yoshino J . SIRT1 selectively exerts the metabolic protective effects of hepatocyte nicotinamide phosphoribosyltransferase. Nat Commun. 2022;13:1074. 35228549 10.1038/s41467-022-28717-7 PMC8885655

[5] Zhang Y Higgins CB Mayer AL Mysorekar IU Razani B Graham MJ . TFEB-dependent induction of thermogenesis by the hepatocyte SLC2A inhibitor trehalose. AU - Zhang, Yiming Autophagy. 2018;14:1959–1975. 10.1080/15548627.2018.1493044 PMC6152536 29996716

[6] Higgins CB Zhang Y Mayer AL Fujiwara H Stothard AI Graham MJ . Hepatocyte ALOXE3 is induced during adaptive fasting and enhances insulin sensitivity by activating hepatic PPARγ. JCI Insight. 2018;3:e120794. 30135298 10.1172/jci.insight.120794 PMC6141168

[7] Mayer AL Higgins CB Feng EH Adenekan O Zhang Y DeBosch BJ . Enhanced hepatic PPARα activity links GLUT8 deficiency to augmented peripheral fasting responses in male mice. Endocrinology. 2018;159:2110–2126. 29596655 10.1210/en.2017-03150 PMC6366533

[8] Seyer P Vallois D Poitry-Yamate C Schütz F Metref S Tarussio D . Hepatic glucose sensing is required to preserve β cell glucose competence. J Clin Invest. 2013;123:1662–1676. 23549084 10.1172/JCI65538 PMC3613916

[9] DeBosch BJ Kluth O Fujiwara H Schurmann A Moley K . Early-onset metabolic syndrome in mice lacking the intestinal uric acid transporter SLC2A9. Nat Commun. 2014;5:4642. 25100214 10.1038/ncomms5642 PMC4348061

[10] Thorens B . GLUT2, glucose sensing and glucose homeostasis. Diabetologia. 2015;58:221–232. 25421524 10.1007/s00125-014-3451-1

[11] McMillan EA Ryu MJ Diep CH Mendiratta S Clemenceau JR Vaden RM . Chemistry-first approach for nomination of personalized treatment in lung cancer. Cell. 2018;173:864–78. e29. 29681454 10.1016/j.cell.2018.03.028 PMC5935540

[12] Cui L Ranalletta M Gorovits N Charron MJ Busik JV de-Mouzon SH . Regulation of hepatic GLUT8 expression in normal and diabetic models. Endocrinology. 2003;144:1703–1711. 12697674 10.1210/en.2002-220968

[13] Adastra KL Frolova AI Chi MM Cusumano D Bade M Carayannopoulos MO . Slc2a8 deficiency in mice results in reproductive and growth impairments. Biol Reprod. 2012;87:49. 22649075 10.1095/biolreprod.111.097675 PMC3431428

[14] Lee H Kim E Shin E-A Shon JC Sun H Kim JE . Crosstalk between TM4SF5 and GLUT8 regulates fructose metabolism in hepatic steatosis. Mol Metab. 2022;58:101451. 35123128 10.1016/j.molmet.2022.101451 PMC8866669

[15] Adekola K Rosen ST Shanmugam M . Glucose transporters in cancer metabolism. Curr Opin Oncol. 2012;24:650–654. 22913968 10.1097/CCO.0b013e328356da72 PMC6392426

[16] DeBosch BJ Chen Z Finck BN Chi M Moley KH . Glucose transporter-8 (GLUT8) mediates glucose intolerance and dyslipidemia in high-fructose diet-fed male mice. Mol Endocrinol. 2013;27:1887–1896. 24030250 10.1210/me.2013-1137 PMC3805847

[17] Debosch BJ Chen Z Saben JL Finck BN Moley KH . Glucose transporter 8 (GLUT8) mediates fructose-induced de novo lipogenesis and macrosteatosis. J Biol Chem. 2014;289:10989–10998. 24519932 10.1074/jbc.M113.527002 PMC4036240

[18] Novelle MG Bravo SB Deshons M Iglesias C García-Vence M Annells R . Impact of liver-specific GLUT8 silencing on fructose-induced inflammation and omega-oxidation. iScience. 2021;24:102071. 33554072 10.1016/j.isci.2021.102071 PMC7856473

[19] Zhang Y Shaikh N Ferey JL Wankhade UD Chintapalli SV Higgins CB . Lactotrehalose, an analog of trehalose, increases energy metabolism without promoting clostridioides difficile infection in mice. Gastroenterology. 2020;158:1402–16. e2. 31838076 10.1053/j.gastro.2019.11.295 PMC7103499

[20] Zhang Y DeBosch BJ . Microbial and metabolic impacts of trehalose and trehalose analogues. Gut Microbes. 2020;11:1475–1482. 32329657 10.1080/19490976.2020.1750273 PMC7524367

[21] Zhang Y Higgins CB Van Tine BA Bomalaski JS DeBosch BJ . Pegylated arginine deiminase drives arginine turnover and systemic autophagy to dictate energy metabolism. Cell Rep Med. 2022;3:100498. 35106510 10.1016/j.xcrm.2021.100498 PMC8784773

[22] Mardones P Rubinsztein DC Hetz C . Mystery solved: Trehalose kickstarts autophagy by blocking glucose transport. Sci Signal. 2016;9:fs2. 26905424 10.1126/scisignal.aaf1937

[23] Schmidt S Joost HG Schurmann A . GLUT8, the enigmatic intracellular hexose transporter. Am J Physiol Endocrinol Metab. 2009;296:E614–E618. 19176349 10.1152/ajpendo.91019.2008

[24] Sunny NE Parks EJ Browning JD Burgess SC . Excessive hepatic mitochondrial TCA cycle and gluconeogenesis in humans with nonalcoholic fatty liver disease. Cell Metab. 2011;14:804–810. 22152305 10.1016/j.cmet.2011.11.004 PMC3658280

[25] Reckzeh ES Waldmann H . Small-molecule inhibition of glucose transporters GLUT-1–4. Chembiochem. 2020;21(1-2):45–52. 31553512 10.1002/cbic.201900544 PMC7004114

[26] Tsuchida T Lee YA Fujiwara N Ybanez M Allen B Martins S . A simple diet- and chemical-induced murine NASH model with rapid progression of steatohepatitis, fibrosis and liver cancer. J Hepatol. 2018;69:385–395. 29572095 10.1016/j.jhep.2018.03.011 PMC6054570

[27] Kraft TE Heitmeier MR Putanko M Edwards RL Ilagan MXG Payne MA . A novel fluorescence resonance energy transfer-based screen in high-throughput format to identify inhibitors of malarial and human glucose transporters. Antimicrob Agents Chemother. 2016;60:7407–7414. 27736766 10.1128/AAC.00218-16 PMC5119023

[28] Sun J Zhang Y Adams JA Higgins CB Kelly SC Zhang H . Hepatocyte Period 1 dictates oxidative substrate selection independent of the core circadian clock. Cell Rep. 2024;43:114865. 39412985 10.1016/j.celrep.2024.114865 PMC11601098

[29] ZhangY HigginsCB TicaS AdamsJA SunJ KellySC . Hierarchical tricarboxylic acid cycle regulation by hepatocyte arginase 2 links the urea cycle to oxidative metabolism. Cell Metab. 2024;36:2069–2085. e8.39116884 10.1016/j.cmet.2024.07.007

[30] Higgins CB Adams JA Ward MH Greenberg ZJ Milewska M Sun J . The tetraspanin transmembrane protein CD53 mediates dyslipidemia and integrates inflammatory and metabolic signaling in hepatocytes. J Biol Chem. 2023;299:102835. 36581203 10.1016/j.jbc.2022.102835 PMC9900517

[31] Zhang Y Sun J Wasserman HD Adams JA Higgins CB Kelly SC . A structure-function analysis of hepatocyte arginase 2 reveals mitochondrial ureahydrolysis as a determinant of glucose oxidation. Cell Mol Gastroenterol Hepatol. 2024;17:801–820. 38280549 10.1016/j.jcmgh.2024.01.016 PMC10966292

[32] De Chiara F Heebøll S Marrone G Montoliu C Hamilton-Dutoit S Ferrandez A . Urea cycle dysregulation in non-alcoholic fatty liver disease. J Hepatol. 2018;69:905–915. 29981428 10.1016/j.jhep.2018.06.023

[33] Kelly SC Higgins CB Sun J Adams JA Zhang Y Ballentine S . Hepatocyte MMP14 mediates liver and inter-organ inflammatory responses to diet-induced liver injury. PNAS Nexus. 2024;3:pgae357. 39282008 10.1093/pnasnexus/pgae357 PMC11393575

[34] Potthoff MJ Inagaki T Satapati S Ding X He T Goetz R . FGF21 induces PGC-1alpha and regulates carbohydrate and fatty acid metabolism during the adaptive starvation response. Proc Natl Acad Sci USA. 2009;106:10853–10858. 19541642 10.1073/pnas.0904187106 PMC2705613

[35] Markan KR Naber MC Ameka MK Anderegg MD Mangelsdorf DJ Kliewer SA . Circulating FGF21 is liver derived and enhances glucose uptake during refeeding and overfeeding. Diabetes. 2014;63:4057–4063. 25008183 10.2337/db14-0595 PMC4238010

[36] Hruz PW Murata H Qiu H Mueckler M . Indinavir induces acute and reversible peripheral insulin resistance in rats. Diabetes. 2002;51:937–942. 11916910 10.2337/diabetes.51.4.937

[37] De Giorgis V Veggiotti P . GLUT1 deficiency syndrome 2013: Current state of the art. Seizure. 2013;22:803–811. 23890838 10.1016/j.seizure.2013.07.003

[38] Abel ED Peroni O Kim JK Kim YB Boss O Hadro E . Adipose-selective targeting of the GLUT4 gene impairs insulin action in muscle and liver. Nature. 2001;409:729–733. 11217863 10.1038/35055575

[39] Kahn BB . Lilly lecture 1995. Glucose transport: pivotal step in insulin action. Diabetes. 1996;45:1644–1654. 8866574 10.2337/diab.45.11.1644

[40] Preitner F Bonny O Laverrière A Rotman S Firsov D Da Costa A . Glut9 is a major regulator of urate homeostasis and its genetic inactivation induces hyperuricosuria and urate nephropathy. Proc Natl Acad Sci USA. 2009;106:15501. 19706426 10.1073/pnas.0904411106 PMC2741280

[41] Toyama T Neuen BL Jun M Ohkuma T Neal B Jardine MJ . Effect of SGLT2 inhibitors on cardiovascular, renal and safety outcomes in patients with type 2 diabetes mellitus and chronic kidney disease: a systematic review and meta-analysis. Diabetes, Obes Metab. 2019;21:1237–1250. 30697905 10.1111/dom.13648

[42] Zezina E Sercan-Alp O Herrmann M Biesemann N . Glucose transporter 1 in rheumatoid arthritis and autoimmunity. Wiley Interdiscip Rev Syst Biol Med. 2020;12:e1483. 32084302 10.1002/wsbm.1483

[43] Kading J Finck BN DeBosch BJ . Targeting hepatocyte carbohydrate transport to mimic fasting and calorie restriction. FEBS J. 2020;288:3784–3798. 32654397 10.1111/febs.15482 PMC8662989

[44] Milewska M Milewski A Wandzik I Stenzel MH . Structurally analogous trehalose and sucrose glycopolymers—Comparative characterization and evaluation of their effects on insulin fibrillation. Polym Chem. 2022;13:1831–1843.

[45] Wada SI Arimura H Nagayoshi M Sawa R Kubota Y Matoba K . Rediscovery of 4-Trehalosamine as a biologically stable, mass-producible, and chemically modifiable trehalose analog. Adv Biol (Weinh). 2022;6:e2101309. 35297567 10.1002/adbi.202101309

[46] Chaix A Zarrinpar A Miu P Panda S . Time-restricted feeding is a preventative and therapeutic intervention against diverse nutritional challenges. Cell Metab. 2014;20:991–1005. 25470547 10.1016/j.cmet.2014.11.001 PMC4255155

[47] Danielson ND Collins J Stothard AI Dong QQ Kalera K Woodruff PJ . Degradation-resistant trehalose analogues block utilization of trehalose by hypervirulent Clostridioides difficile. Chemical Communications. 2019;55:5009–5012. 30968891 10.1039/c9cc01300h PMC6499371

[48] Satapati S Kucejova B Duarte JA Fletcher JA Reynolds L Sunny NE . Mitochondrial metabolism mediates oxidative stress and inflammation in fatty liver. J Clin Invest. 2015;125:4447–4462. 26571396 10.1172/JCI82204 PMC4665800

[49] McCommis Kyle S Finck Brian N . Mitochondrial pyruvate transport: A historical perspective and future research directions. Biochem J. 2015;466:443–454. 25748677 10.1042/BJ20141171 PMC4464838

[50] Simon J Nuñez-García M Fernández-Tussy P Barbier-Torres L Fernández-Ramos D Gómez-Santos B . Targeting hepatic glutaminase 1 ameliorates non-alcoholic steatohepatitis by restoring very-low-density lipoprotein triglyceride assembly. Cell Metab. 2020;31:605–22. e10. 32084378 10.1016/j.cmet.2020.01.013 PMC7259377

