# Supplementary material for: Targeting hepatocyte-specific SLC2A8 blocks hepatic steatosis and dissociates TCA cycle flux inhibition from glutamine anaplerosis
Source: Hepatol Commun. 2025 Sep 22;9(10):e0810. doi: 10.1097/HC9.0000000000000810 (PMC12456581; doi:10.1097/HC9.0000000000000810)

Supplementary Figure 1. TG content and inflammatory gene expression in Glut1- and Glut2-deficient hepatocytes treated with or with FA + LPS *in vitro*. Enzymatic-colorimetric triglyceride quantification and qRT-PCR analysis of inflammatory gene expressions in response to 24hr FA and LPS treatment. *, **, ***, and **** represent P < 0.05, < 0.01, < 0.001, and P < 0.0001, respectively, by 2-way ANOVA with Sidak's post hoc test.

Supplementary Figure 1

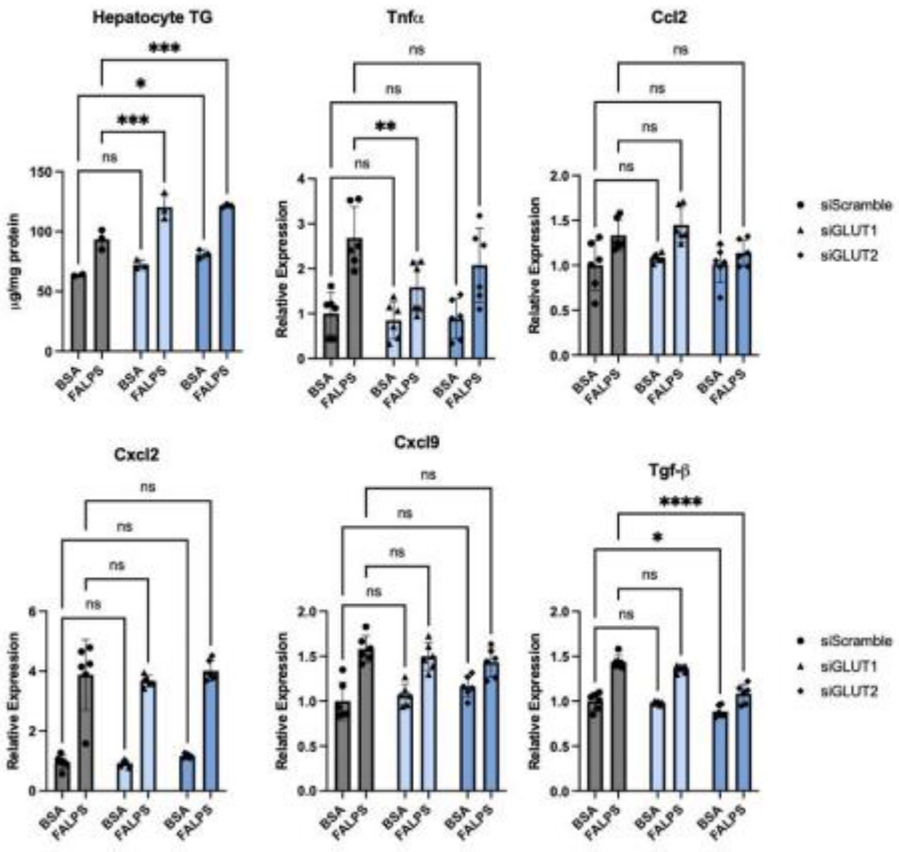

Supplementary Figure 2. KEGG pathways comparing livers from GLUT8^{LKO} and GLUT8^{WT} mice fed MASH diet. Pathway analyses demonstrating the most highly significant down- and up-regulated KEGG pathways.

Supplementary Figure 2

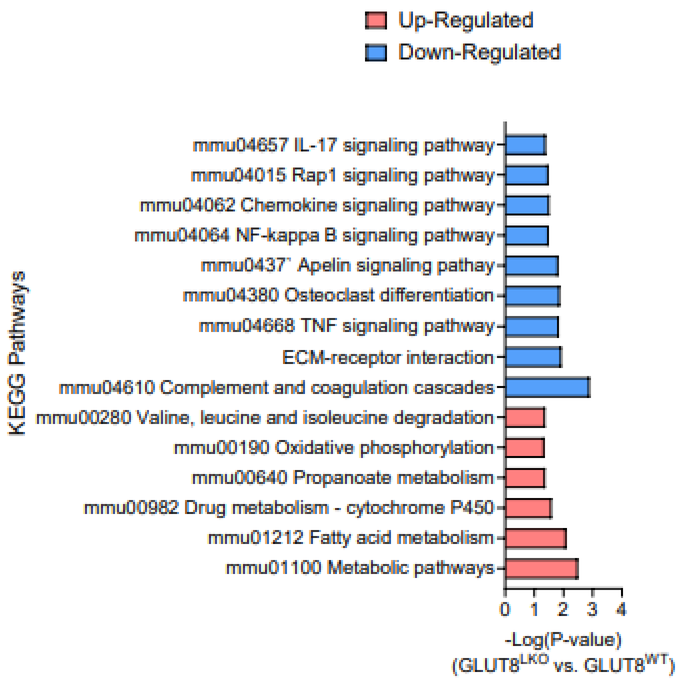

Supplementary Figure 3. GLUT8 selectivity of P20. Radiolabeled carbohydrate uptake was quantified in 293 cells overexpressing either GLUT1, GLUT3, GLUT4 or GLUT5 in the presence or absence of escalating [P20]. Calculated IC_{50} for each isoform and substrate is shown for each inhibition curve.

Supplementary Figure 3

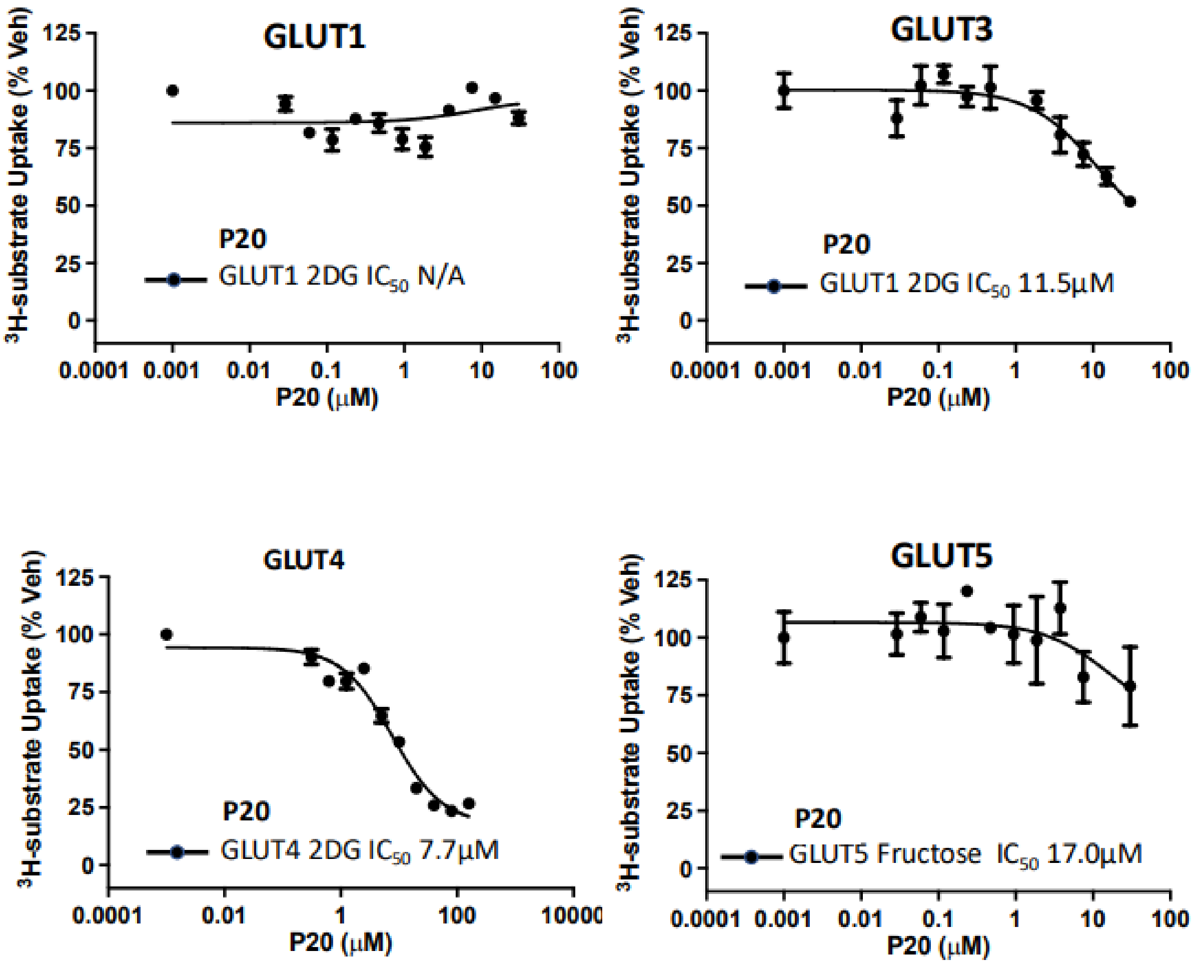

Supplement: Supplementary file 1 [file hc9-9-e0810-s001.pdf]
